# Data from static and dynamic mechanical tests of different isomers of amine cured multifunctional epoxy resins

**DOI:** 10.1016/j.dib.2018.05.125

**Published:** 2018-05-25

**Authors:** Roderick Ramsdale-Capper, Joel P. Foreman

**Affiliations:** Department of Materials Science and Engineering, Sir Robert Hadfield Building, University of Sheffield, Sheffield S1 3JD, UK

## Abstract

Data from gas pycnometry, static compressive stress-strain and dynamic mechanical analysis are presented for a series of aromatic amine cured epoxy resins. Samples are prepared and tested which consist of para-para, para-meta, meta-para and meta-meta isomers of the epoxy and amine phenylene ring respectively. The density data consists of 25 measurements on 3 separate samples of each of the 4 sample types. The static compressive stress–strain data consists of at least 5 tests on separate samples of each of the 4 samples types. The dynamic mechanical analysis data consists of multiple frequency, loss tangent measurements of at least 6 separate samples of each of the 4 sample types. The data is interpreted in the accompanying research article, ‘Internal antiplasticisation in highly crosslinked amine cured multifunctional epoxy resins’ (Ramsdale-Capper and Foreman, submitted for publication) [1].

**Specifications Table**

**Table:** 

Subject area	*Materials Science & Engineering*
More specific subject area	*Polymers, Thermosets, Materials Characterisation*
Type of data	*Spreadsheets containing data and plots*
How data was acquired	*Gas pycnometry (Micromeritics AccupycII 1340), static compressive mechanical testing (Instron 5582), dynamic mechanical analysis (Perkin Elmer DMA8000).*
Data format	*Analysed:*
	1. *Gas pycnometry data (volume) converted to density* 2. *Static compressive mechanical testing data (load, displacement) converted to stress-strain and a compliance correction was used* 3. *Dynamic mechanical analysis data (storage modulus, loss modulus) converted to loss tangent (tanδ)*
Experimental factors	*Samples were cured using an epoxy:amine ratio of 100:36. Amine was dissolved in epoxy at 120 °C and degassed at 100 °C before casting. The cure cycle was 100 °C preheat temperature, ramp to 130 °C at 2 °C/min, dwell at 130 °C for 1 h, ramp to 200 °C at 2 °C/min, dwell at 200 °C for 2 h and finally ramp to 25 °C at 2 °C/min. Specimens prepared as described in**[1]*.
Experimental features	*Samples were stored in a desiccator over phosphorous pentoxide between casting and testing. Samples were also subject to weighing and vacuum drying before testing.*
Data source location	*Department of Materials Science, University of Sheffield, Sheffield, UK.*
Data accessibility	*Data in article.*

**Value of the data**

• The data contained in the article provides the community with individual data for each test performed on a series of resin samples, allowing readers to perform their own statistical analyses potentially providing new insights into the material performance. In particular, density measurements over multiple cycles, compressive stress-strain data and multiple frequency storage modulus and loss tangent for each sample type.
• The data provides benchmarks for this type of high performance epoxy resins used in the aerospace industry and allows readers to compare their values to those presented here where performance versus weight ratios are key.
• The data provides measurements of samples with a variety of different isomeric components which allows readers to tailor the properties of resins accordingly.

## 1 Data

1. Pycnometry. The spreadsheet contains the calculated densities of each sample type. The samples contain para-para, para-meta, meta-para or meta-meta substituted phenylene rings in the epoxy or amine respectively. 25 measurements are taken on 3 separate samples giving 75 measurements for each sample type and a total of 300 measurements overall.
2. Static compressive stress-strain. The spreadsheet contains the calculated applied stresses and calculated experience strains for each sample type. The samples contain para-para, para-meta, meta-para or meta-meta substituted phenylene rings in the epoxy or amine respectively. At least 5 tests are done on each sample type giving a total of at least 20 stress-strain curves. The data is plotted as a stress-strain curve for each test.
3. Dynamic mechanical analysis. The spreadsheet contains the calculated loss tangent values for each sample type. The samples contain para-para, para-meta, meta-para or meta-meta substituted phenylene rings in the epoxy or amine respectively. The tests are run between− 160 °C and 300 °C and at 4 different frequencies (1, 5, 10, 50 Hz). At least 6 tests are done on each sample type giving a total of 96 loss tangent plots. These are combined onto 24 multi-frequency plots.
4. Raw data is in the attached spreadsheets and summaries are given in the experimental design, materials and methods section of this article.

## 2 Experimental design, materials and methods

The epoxy resin isomers used are triglycidyl *p*-aminophenol (TGPAP) and triglycidyl *m*-aminophenol (TGMAP) (supplied as Araldite MY0510 and Araldite MY0610 respectively by Huntsman Advanced Materials). The amine isomers used are 4,4’diaminodiphenylsulphone (44DDS) (supplied by Sigma Aldrich) and 3,3′diaminodiphenylsulphone (33DDS) (supplied as Aradur 9719-1 by Huntsman Advanced Materials). When cured, four different epoxy resin sample types are created, denoted as TGPAP/44DDS, TGPAP/33DDS, TGMAP/44DDS and TGMAP/33DDS. The four sample types represent para-para, para-meta, meta-para and meta-meta combinations of the phenylene ring structural isomers respectively.

For this study an epoxy rich epoxy:amine ratio of 100:36 is used. The uncured epoxy is initially heated to 60 °C to reduce its viscosity, after which the DDS is added. The temperature of the mixture is increased to 120 °C and mechanically stirred for approximately 30 minutes until the DDS has dissolved producing a clear homogeneous solution. The solution is then placed into a vacuum oven at 100 °C to degas and then poured into a preheated mould prior to cure. The cure cycle was 100 °C preheat temperature for the mould, ramp to 130 °C at 2 °C/min, dwell at 130 °C for 1 h, ramp to 200 °C at 2 °C/min, dwell at 200 °C for 2 h and finally ramp to 25 °C at 2 °C/min. The samples were ground top and bottom to remove a thin layer to prevent issues with surface oxidation or remnants of the release agent affecting the resin properties. Further information regarding experimental procedure and sample preparation can be found in ‘Internal Antiplasticisation in Highly Crosslinked Amine Cured Multifunctional Epoxy Resins’ [1].

A Perkin Elmer DMA8000 was used in single cantilever mode to measure the dynamic response in each sample as a result of applying a sinusoidal force at 1, 5, 10 & 50 Hz. Samples were cut into rectangular coupons measuring 30 mm × 10 mm × 1.6 mm. The samples were scanned from − 160 °C to 300 °C at a heating rate of 3 °C/min using a strain amplitude of 0.05 mm. Table 1, Table 2 express the analysed data of the glass transition temperature (*T*_g_) and the beta transition temperature (*T*_β_).

**Table 1:** *T*_g_ peak values of the loss tangent curve for each of the epoxy resin variants. Numbers represent the sample number analysed.

	*T*_g_/°C							
Sample Type	1	2	3	4	5	6	Average	SD
TGPAP/44DDS	270	271	270	270	270	270	270	1
TGPAP/33DDS	232	231	230	231	231	231	231	1
TGMAP/44DDS	237	237	237	237	236	237	237	1
TGMAP/33DDS	212	212	212	211	211	212	212	1

**Table 2:** *T*_β_ peak values of the loss tangent curve for each of the epoxy resin variants. Numbers represent the sample number analysed.

	*T*_β_/°C							
Sample Type	1	2	3	4	5	6	Average	SD
TGPAP/44DDS	− 38	− 40	− 37	− 35	− 36	− 38	− 37	2
TGPAP/33DDS	− 47	− 42	− 41	− 42	− 43	− 40	− 43	2
TGMAP/44DDS	− 39	− 40	− 37	− 37	− 39	− 39	− 39	1
TGMAP/33DDS	− 42	− 44	− 43	− 41	− 45	− 43	− 43	1

A Micromeritics AccupycII 1340 helium gas pycnometer was used to measure the volume of all samples by measuring the amount of gas displaced at 19.00 psi. Testing consisted of 25 cycles on three 10 mm diameter samples of each type at 27 °C. The density is then calculated after measuring the mass on a four point balance. The data from this test is expressed within Table 3.

**Table 3:** Analysed density data from a helium gas pycnometer of the epoxy resin variants TGPAP/44DDS, TGPAP/33DDS, TGMAP/44DDS and TGMAP/33DDS.

	Density/g cm^−3^			
Sample Type	Sample 1	Sample 2	Sample 3	Average
TGPAP/44DDS	1.3139 ± 0.0006	1.3105 ± 0.0007	1.3102 ± 0.0008	1.3115 ± 0.0018
TGPAP/33DDS	1.3161 ± 0.0010	1.3123 ± 0.0004	1.3135 ± 0.0005	1.3140 ± 0.0017
TGMAP/44DDS	1.3240 ± 0.0010	1.3260 ± 0.0006	1.3234 ± 0.0006	1.3245 ± 0.0014
TGMAP/33DDS	1.3256 ± 0.0011	1.3266 ± 0.0007	1.3241 ± 0.0007	1.3254 ± 0.0014

An Instron 5582 tensometer was used to measure the static compressive load-response properties of the samples. Compression samples were cut into cylinders of 10 mm diameter which were then machined to 10 mm lengths with parallel faces. Five samples of each resin variant were tested at a strain rate of 1 mm/min at 30 °C. The analysed data for the four sample variants are expressed within Table 4, Table 5, Table 6, Table 7.

**Table 4:** Analysed compressive properties of TGPAP/44DDS.

TGPAP/44DDS				
Sample no.	Young׳s Modulus/GPa	Yield stress/MPa	Strain to failure	UTS/MPa
1	3.19	/	0.402	242
2	3.34	/	0.396	238
3	3.15	/	0.343	220
4	3.51	/	0.405	245
5	3.37	/	0.412	249
Average	3.31	/	0.392	239
SD	0.14	/	0.028	11

**Table 5:** Analysed compressive properties of TGPAP/33DDS.

TGPAP/33DDS				
Sample no.	Young׳s Modulus/GPa	Yield Stress/MPa	Strain to failure	UTS/MPa
1	3.77	190	0.409	236
2	3.59	188	0.379	219
3	3.72	188	0.383	223
4	3.72	188	0.415	236
5	3.61	190	0.407	235
Average	3.68	189	0.399	230
SD	0.07	1	0.015	8

**Table 6:** Analysed compressive properties of TGMAP/44DDS.

TGMAP/44DDS				
Sample no.	Young׳s Modulus/GPa	Yield stress/MPa	Strain to failure	UTS/MPa
1	3.92	206	0.383	206
2	4.31	208	0.516	226
3	4.14	210	0.583	250
4	4.13	208	0.452	208
5	4.20	208	0.458	214
Average	4.14	208	0.478	221
SD	0.14	1	0.075	18

**Table 7:** Analysed compressive properties of TGMAP/33DDS.

TGMAP/33DDS				
Sample no.	Young׳s Modulus/GPa	Yield stress/MPa	Strain to failure	UTS/MPa
1	4.39	214	0.563	234
2	4.46	214	0.624	266
3	4.59	214	0.413	214
4	4.54	216	0.575	244
5	4.44	211	0.308	211
Average	4.48	214	0.496	234
SD	0.08	2	0.132	23
