# Supplementary material for: Data from static and dynamic mechanical tests of different isomers of amine cured multifunctional epoxy resins
Source: Data Brief. 2018 May 25;19:992–6. doi: 10.1016/j.dib.2018.05.125 (PMC5997956; doi:10.1016/j.dib.2018.05.125)
Supplement: Supplementary file 1 — Supplementary material [file mmc1.docx]

Conflict of Interest

None.
